# Selected Papers from the Second International Online Conference on *Nanomaterials*

**DOI:** 10.3390/nano12030302

**Published:** 2022-01-18

**Authors:** Ana M. Díez-Pascual, Antonio Di Bartolomeo, Guanying Chen

**Affiliations:** 1 Universidad de Alcalá, Facultad de Ciencias, Departamento de Química Analítica, Química Física e Ingeniería Química, Ctra. Madrid-Barcelona, Km. 33.6, 28805 Madrid, Spain; 2 Physics Department, University of Salerno, via Giovanni Paolo II 132, 84084 Fisciano, Italy; adibartolomeo@unisa.it; 3 School of Chemistry and Chemical Engineering, Harbin Institute of Technology, Harbin 150090, China; chenguanying@hit.edu.cn

Nanomaterials have gained eminence in technological developments due to their tunable physical, chemical, and biological properties, such as wettability, electrical and thermal conductivity, magnetism, light absorption and emission, catalytic activity, and so forth, leading to devices with improved performance compared to their microscopic counterparts. They have at least a dimension in the 0.1 to 100 nm range, and are classified based on their size, composition, shape, and origin. Thus, four main categories can be distinguished based on their nature [1]: (1) carbon-based nanomaterials, which are found in morphologies such as ellipsoids or spheres, hollow tubes, or ultrathin sheets; fullerenes (C_60_), carbon nanotubes (CNTs), carbon nanofibers, carbon black, graphene (G), and its derivatives graphene oxide (GO) and reduced graphene oxide (rGO) are in this category [2,3]. (2) Inorganic-based nanomaterials, which include metal [4] and metal oxide nanoparticles, such as TiO_2_, SnO and ZnO [5,6,7], and semiconductors, such as silicon, III-V compounds, transition metal dichalcogenides, Xenes, etc. [8]. (3) Organic-based nanomaterials, which include dendrimers, micelles, liposomes and polymer nanoparticles. (4) Composite-based nanomaterials, with one phase on the nanoscale dimension that can either combine nanoparticles with other nanoparticles or other structures such as metal–organic frameworks. The composites may be any combinations of carbon-, metal-, or organic-based nanomaterials with any form of metal, ceramic, or polymer bulk materials [9,10].

This Special Issue collects fourteen selected papers from the Proceedings of the second International Online Conference on Nanomaterials, held on 15–30 November 2020 in Sciforum (https://sciforum.net/conference/IOCN2020), an online platform for scholarly e-conferences and discussion groups that presents the most recent advances in the area of nanomaterials.

The articles present very different types of nanomaterials, such as gold nanorods [4], WTe_2_ nanocrystals [11], CaCO_3_ nanoparticles [12], ferromagnetic nanoparticles [13], CuO nanocrystals and single-walled carbon nanotubes [14], graphene [3], and GO derivatives [15].

Different techniques have been applied to characterize these nanomaterials, including X-ray diffraction, optical profilometry, cyclic voltammetry, IR and Raman spectroscopies, ellipsometry, dynamic light scattering (DLS), contact angle measurements, N_2_ adsorption-desorption experiments, atomic force microscopy (AFM), ultraviolet–visible (UV–VIS), fluorescence, X-ray photoelectron (XPS) and electrochemical impedance (EIS) spectroscopies, scanning and transmission electronic microscopies (SEM and TEM).

Moreover, the developed materials can be used for a wide range of applications: (1) drug delivery systems to deliver gefitinib (GEF) and paclitaxel (PTXL) to treat breast cancer [12] or to release antibiotics such as ciprofloxacin, as well as some other small model drug compounds [16]; (2) materials for the removal of toxic contaminants in water, such as As, Mn, Cr, and Cd from ground water and natural water conditions [17]; (3) membrane catalysis with specific parameters [18]; (4) quaternary memory systems for applications in data storage and processing [19]; (5) electrochromic devices, which control optical properties such as optical transmission, absorption, reflectance, and/or emittance in a continual but reversible manner on the application of voltage [20]; (6) flexible electrodes with high optical transparency, low electrical resistance, and mechanical bending stability for use in flexible polymer-dispersed liquid crystal (PDLC) structures (7) in optoelectronic devices as well as outdoor displays, projection displays, switchable privacy glasses, energy-saving windows, light shutters and so forth; and (8) hole transport layers in inverted perovskite solar cells.

The articles published in the Special Issue of the second International Online Conference on Nanomaterials (IOCN 2020) highlight the important role of nanomaterials for the progress of science, technology, and human healthcare. We expect that more and more researchers can join the open access ICON forum in the future to promote the development of nanoscience and nanotechnology.

## Data Availability

Data sharing is not applicable to this article as no new data were created or analyzed in this study.
